# Pulmonary Artery Pseudoaneurysm (PAA) in a Patient With Advanced Lung Cancer After Radiotherapy: A Potentially Fatal Complication

**DOI:** 10.1002/rcr2.70326

**Published:** 2025-08-21

**Authors:** Josephine Coffey, Ayan Sabih

**Affiliations:** ^1^ Institute of Respiratory Waikato Hospital Hamilton New Zealand

**Keywords:** massive pulmonary haemorrhage, pulmonary artery pseudoaneurysm, Radiotherapy

## Abstract

Pulmonary artery pseudoaneurysms (PAAs) are exceedingly uncommon. It is a life‐threatening diagnosis, and often associated with poor long‐term prognosis. We report a case of PAA in a 74‐year‐old man presenting with severe respiratory failure and haemoptysis with a history of advanced lung cancer. Though the intent was to manage the PAA with coil embolisation, this was unsuccessful and the patient passed away 72 h after hospital admission. There is limited evidence for treatment of PAA, and once a patient is symptomatic, has a high mortality.

## Graphical abstract

We report a case of pulmonary artery pseudoaneurysm (PAA) in a 74‐year‐old man presenting with severe respiratory failure and haemoptysis with a history of advanced lung cancer. Though the intent was to manage the PAA with coil embolisation, this was unsuccessful and the patient passed away 72 h after hospital admission. There is limited evidence for treatment of PAA, and once a patient is symptomatic, has a high mortality.

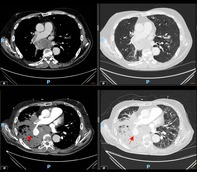

## 1 Introduction

A pseudoaneurysm is a life‐threatening focal dilation of a blood vessel and differs from an aneurysm in that it does not involve all layers of the blood vessel wall, having a higher risk of rupture than an aneurysm. There is very limited data on the formation of pulmonary artery pseudoaneurysms (PAAs), though it has been suggested that structural changes in elastin and collagen under an increase in pressure, lead to dilation. It is thought to be the cause of haemoptysis in less than 10% of cases [1, 2]^.^ The associated mortality of rupture is 50%–100%, with rupture occurring in 87% of cases if left untreated [1, 3].

## 2 Case Report

**FIGURE 1:**
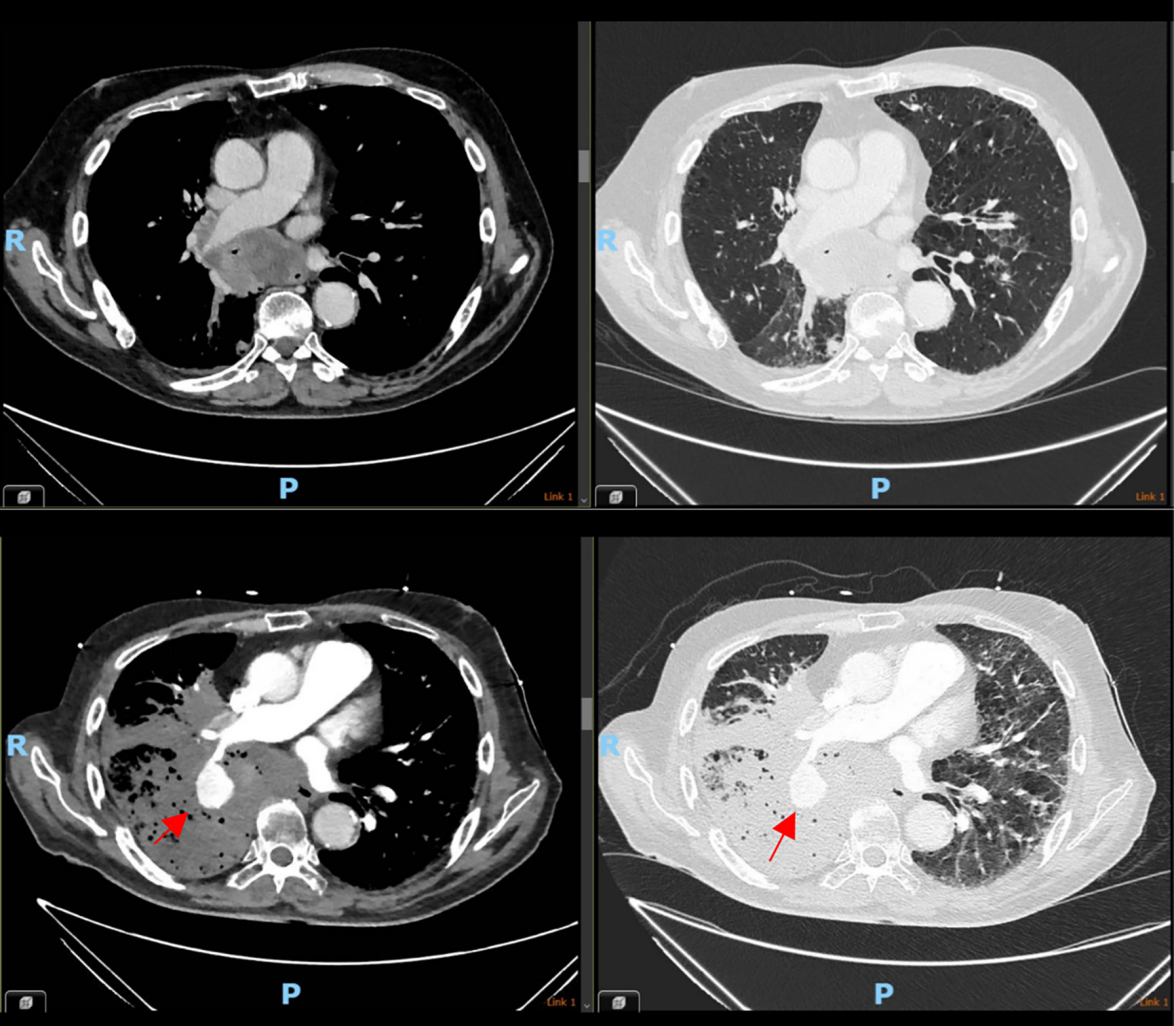
Comparison between the CT chest of the patient in February vs. April, demonstrating a new large pulmonary artery pseudoaneurysm.

Our patient, a 74‐year‐old gentleman had initially been referred to the local respiratory department after presentation to a primary care physician for dry cough and hypercalcaemia in February 2025. CT of the chest in March 2025 (Figure 1) demonstrated bilateral pulmonary nodules, a large centrally necrotic mass encasing the right main bronchus, sub‐cardinal mediastinal and supraclavicular lymphadenopathy and extensive emphysematous change. His past medical history included a diagnosis of chronic obstructive pulmonary disease (COPD) with lung function tests in March 2025 demonstrating FEV1 of 2.08L/73% predicted, FVC 3.59L/92% predicted, FEV1/FVC of 0.58, and reduced gas transfer of DLCO 44% and current smoker with 13‐year‐pack history, prostate cancer (in remission) and bilateral total hip replacement (indication of osteoarthritis). He underwent flexible bronchoscopy, which demonstrated a locally advanced tumour with near obstruction of the right main stem from the circumferential tumour infiltration, extending to the carina. Biopsy confirmed squamous cell carcinoma (PDL1 expression > 50%). The case was discussed at the tumour board meeting, and radiologically staged as T4N3M1b—stage IVA (TNM Staging 9th edition). The patient's performance status was preserved at ECOG 0. Due to the risk of airway compromise, he was referred to the radiation oncology service for palliative radiotherapy and completed this treatment (30 Gy/10 fractions) on 11 April 2025, with no documented toxicity complications upon review in the clinic of 24 April 2025. The patient had also been referred to the medical oncology service for systemic therapy, and had been waiting for the clinic appointment at the time of presentation to hospital.

The patient presented acutely to the hospital on the 26 April with fever, dyspnoea, hypoxia (saturation 92% on air), tachypnoea, minor volume haemoptysis (reported as less than 50 mL fresh blood), tachycardia with atrial fibrillation and hypotension. He was alert and orientated and initially resuscitated in the Emergency Department. Lab investigations showed anaemia (Hb 100 g/L), normal white cell count (WBC 5.26 × 10^9^/L and neutrophils 4.98 × 10^9^/L), mild hyponatraemia (Na 129 mmol/L), normal renal function (creatinine 87 μmol/L, and urea 6.3 mmol/L), normal troponins (20 ng/L), and raised inflammatory markers (CRP 400 mg/L). Arterial blood gas (ABG) on FiO_2_ of 80% showed pH 7.4, pO₂ 10.0 kPa, pCO₂ 3.8 kPa, bicarbonate 22.7 mmol/L, and a normal lactate (1.4 mmol/L). Blood cultures and sputum cultures were obtained (subsequently found as showing no pathogenic organisms cultured). A bedside chest x‐ray demonstrated right middle and lower zone consolidation and he was started on broad spectrum antibiotics (piperacillin/tazobactam) for chest sepsis. His blood pressure was supported by metaraminol. The rationale for metaraminol for this patient's management of shock was predominantly due to hospital protocol of high dependency unit (HDU) patients in this unit. He was given nebulised and intravenous tranexamic acid for haemoptysis.

**FIGURE 2:**
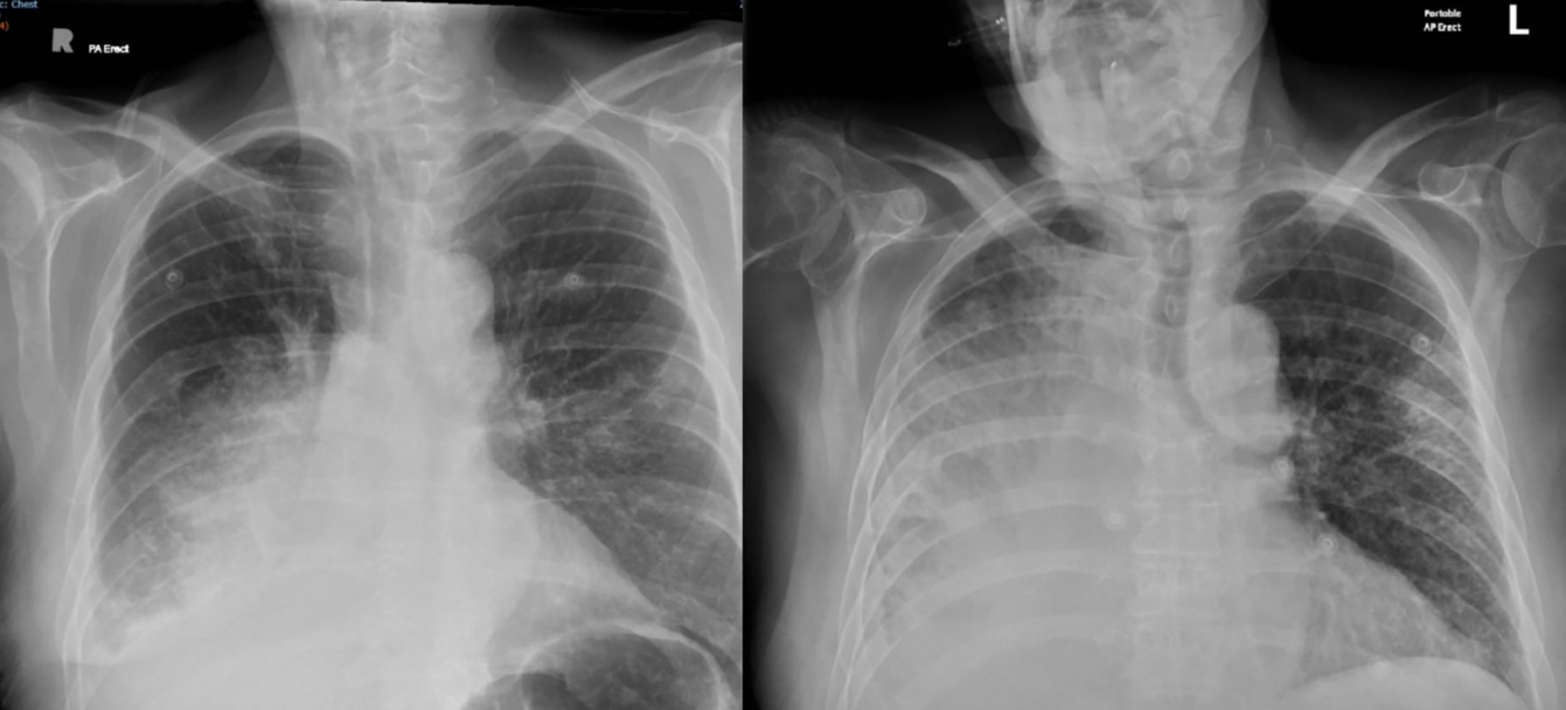
Demonstration of chest x‐ray progression, initial presentation with haemopytsis vs. 48 h later.

He underwent a pulmonary arterial phase CT of the thorax (Figure 2), which demonstrated a new finding of a 3.2 cm pseudoaneurysm arising from the right lower lobe lobar artery with extensive surrounding consolidation involving nearly the entire right lower and middle lobes, in keeping with pulmonary haemorrhage.

The patient was admitted to the HDU for ongoing care and management of the PAA, chest sepsis, atrial fibrillation and hypotension. The cardiothoractic surgeons were consulted, who did not think a surgical intervention was feasible and recommended an interventional radiology (IR) approach. IR reviewed the images and offered to assist with endovascular coiling of the aneurysm. Given the severity of his respiratory failure and co‐morbidites of the patient, multidisciplinary input from intensive care, anaesthesia, respiratory medicine and interventional radiology was required due to the poor prognosis.

Unfortunately, the patient's condition deteriorated prior to the procedure with worsening haemodynamic instability and hypoxic respiratory failure requiring escalating vasopressor support (maximum amount of vasopressor support for hospital HDU is metraminol 10 mg/h in this unit) and FiO_2_ 100%. An updated CXR showed worsening opacification of the right hemithorax. Despite best supportive cares, the clinical picture did not improve to the point that he could safely undergo the IR procedure. Palliative care input was sought, and the patient passed away within 72 h of presentation to hospital. This patient was comorbid and it is likely that PAA only contributed to his death. The severity of respiratory failure was multi‐factorial and his death was likely due to the burden of system disease from his lung cancer, COPD, atrial fibrillation and pneumonia.

## 3 Discussion

PAA though exceedingly uncommon, is a life‐threatening diagnosis, and often associated with poor long‐term prognosis, particularly in those patients presenting with haemoptysis [1]. Pseudoaneurysms do not involve all layers of the artery, like true aneurysms; this makes them a higher risk for rupture [2, 4, 5]. Once ruptured, mortality has been reported to be between 50%–100% [3].

PAA can be congenital or acquired, with causes being trauma, infection, vasculitis and iatrogenic formation (placement of pulmonary catheter, right heart catheter, intubation, lobectomy and radiation) [1, 2, 4]. In particular, metastatic primary lung cancer can lead to PAA where there is often associated erosion in the pulmonary arteries. With these patients, there is a survival rate of 46%–67% in the first 1–3 months after embolotherapy. Diagnosis is usually made though contrast enhanced CT; MRI can also be used as an imaging modality [2, 4, 5].

There is a particular lack of evidence for patients with PAA potentially caused by radiation; however, a literature review of 10 cases suggested that the majority of these patients were middle‐aged or older males that had presented with haemoptysis, like our patient. This highlights PAA as an important differential. Another highlight from this particular study, was that there was a wide range between time of received radiation therapy and the presentation with haemoptysis, from 1 week to 7 years [5]. However, there was no proven causal effect between radiation and the development of PAA in this study.

Pulmonary artery hypertension (PAH), is another important cause of PAA and thought to occur due to increased pressure, causing remodelling and dilation of the artery [2]^.^ Though PAA has a high mortality rate, it cannot be overlooked that this patient had advanced lung cancer and sepsis, and his death and deterioration was multifactorial.

The optimal treatment for PAA is uncertain with no clear management. It has been suggested that once a patient is symptomatic, a conservative approach would not be appropriate due to the high mortality. Treatment options include surgery—lobectomy, aneyrsmectomy and pneumonectomy and endovacsular embolisation with the occlusion of the inflow, outflow and sac of the PAA [1, 3]. Coil embolisation, in the appropriate location, can be a minimally invasive procedure, which can potentially allow for preservation of the surrounding arteries, but with risk of rupturing of the PAA and risk of shunting. Other interventional approaches include vascular plugs, stent‐assisted coil embolisation and stent graphs [3]. These procedures carry their own risks in co‐morbid patients.

Given the limited evidence base and the complexity of these cases, multidisciplinary team discussion is critical in forming an individual treatment plan for high‐risk patients.

## Consent

The authors declare that written informed consent was obtained for the publication of this manuscript and accompanying images using the consent form provided by the Journal.

## Conflicts of Interest

The authors declare no conflicts of interest.

## Data Availability

Data sharing not applicable to this article as no datasets were generated or analysed during the current study.
